# Estimated cut-off values for pemphigus severity classification according to pemphigus disease area index (PDAI), autoimmune bullous skin disorder intensity score (ABSIS), and anti-desmoglein 1 autoantibodies

**DOI:** 10.1186/s12895-020-00105-y

**Published:** 2020-10-31

**Authors:** Farnam Mohebi, Soheil Tavakolpour, Amir Teimourpour, Roja Toosi, Hamidreza Mahmoudi, Kamran Balighi, Narges Ghandi, Maryam Ghiasi, Pedram Nourmohammadpour, Vahideh Lajevardi, Robabeh Abedini, Armaghan Azizpour, Maryam Nasimi, Maryam Daneshpazhooh

**Affiliations:** 1 Autoimmune Bullous Diseases Research Center, Tehran University of Medical Sciences, Razi Hospital, Vahdate-Eslami Square, Tehran, 11996 Iran; 2 Non-Communicable Diseases Research Center, Endocrinology and Metabolism Population Sciences Insitute, Tehran University of Medical Sciences, Tehran, Iran; 3 Department of Biostatistics, School of Public Health, Tehran University of Medical Sciences, Tehran, Iran

**Keywords:** Pemphigus, Pemphigus disease area index, Autoimmune bullous skin disorder intensity score, PDAI, ABSIS, Anti-desmoglein

## Abstract

**Background:**

Pemphigus is a potentially fatal disease if left untreated. Valid scoring systems and defined cut-off values for classification of patients would help with better management through specified pharmaceutical and non-pharmaceutical treatments.

**Methods:**

In this study, pemphigus patients who were receiving immunosuppressive treatments and had recent disease relapse were recruited for examination of pemphigus disease area index(PDAI), autoimmune bullous skin disorder intensity score (ABSIS), physician global assessment (PGA), autoimmune bullous disease quality of life (ABQoL), anti-desmoglein 1 (anti-Dsg1), and anti-Dsg3 autoantibody titers from December-2017 to February-2018. Cut-off values were estimated using model-based clustering classification and the 25th and 75th percentiles approach, performed separately for the exclusive cutaneous, exclusive mucosal, and mucocutaneous groups.

**Results:**

In the 109 included patients, the 25th and 75th percentiles cut-offs were 6.2 and 27 for PDAI score, and 4 and 29.5 for ABSIS score. The model-based analysis resulted in two groups (cut-point:15) for PDAI score, and three groups (cut-points:6.4 and 31.5) for ABSIS score. The groups were significantly different for the PDAI, ABSIS, PGA, and ABQoL values. Based on anti-Dsg1 autoantibody values, the model-based analysis cut-point was 128 and the 25th and 75th percentiles cut-offs were 98 and 182. Anti-Dsg3 autoantibody values did not differentiate between pemphigus severity classes.

**Conclusions:**

Estimated cut-off values based on the anti-Dsg1 level, PDAI, and ABSIS scoring systems could be used to classify patients into different severity grades for better management and prognosis.

## Background

Pemphigus is a rare, but severe autoimmune-blistering disease, which involves skin and/or mucous membranes. In addition to the non-classical forms of pemphigus, there are two major types, including pemphigus vulgaris (PV) and pemphigus foliaceus (PF) [1]. Like other autoimmune conditions, it is caused by the development of aberrant immune responses, with largely unknown etiology, and is associated with both environmental triggers and genetic factors. However, the autoantibodies against desmosomal cadherins desmogleins, including anti-desmoglein 1 (anti-Dsg1) and anti-Dsg3 are widely debated as the main factors in disease development. These autoantibodies result in acantholysis, blisters, and erosions of the skin and the mucous membrane of the mouth, nose, throat, eyes, or genitals [2].

During recent years, we have witnessed significant improvements in the management of pemphigus patients, and notably, some scoring systems have been developed to measure the severity of the disease to help with better management of the patients by categorizing them in a standard manner. Pemphigus patients are sometimes advised to be treated as aggressive as possible, due to the unpredictable clinical course the disease and the probability of exacerbating into the explosive full-blown stage. However, categorizing patients based on severity could be useful for better comparison of different studies and treatment responses and also more precise designing of clinical trials and patient recruitment. Besides, it could be utilized in monitoring the disease progress, clinical assessment of relapses, and evaluating the effectiveness of treatment. Thus, all the aforementioned notions necessitate a universal, reliable and valid tool for categorizing patients based on their severity.

Although some simple systems, like the Physician Global Assessment (PGA), has been most validated for few dermatology diseases, such as acne and psoriasis [3], it has not been accepted as a gold standard system for assessing the severity of pemphigus. Much effort has been made for development of a specific and sensitive scoring systems, able to present pemphigus severity, which have been resulted in introduction of Pemphigus Disease Area Index (PDAI), Autoimmune Bullous Skin Disorder Intensity Score (ABSIS), Pemphigus Vulgaris Activity Score (PVAS), and Harman’s scoring systems [4]. Among them, PDAI, developed by the International Pemphigus Definitions Group in 2009 [5], and the ABSIS, proposed by the German Blistering Disease Group in 2007 [6], seem to be the most sensitive and reliable systems for evaluation of pemphigus severity [7]. However, there is no consensus for categorizing tools and commonly utilized cut-off values of patients based on the severity of the disease for categorizing patients as well, albeit their established robustness [8].

Therefore, we aimed to reevaluate cut-off values for PDAI and ABSIS scores in Iranian pemphigus patients with exclusive cutaneous involvement, exclusive mucosal involvement, with mucocutaneous involvement, and total scores, alongside with possible valid cut-off points for anti-Dsg1 and anti-Dsg3 values.

## Methods

### Included patients

This prospective study was conducted in the Autoimmune Bullous Diseases Research Center, Razi Hospital, Tehran University of Medical Sciences from December 2017 to February 2018. Patients were included if they had diagnosed PV made by dermatologist based on clinical examination, histology findings, direct immunofluorescence examination, and presentence of circulating autoantibodies against the Dsg1 and/or Dsg3, and were receiving treatment and experiencing a recent disease relapse, defined as the appearance of ≥3 new lesions in the month that do not heal spontaneously [9].

### Informed consent

Written informed consent was obtained from all individual participants included in the study.

### Severity assessments

PDAI activity score, developed by the International Pemphigus Definitions Group in 2009 [5], can range from 0 to 250 scores; cutaneous involvement consisting of 120 for skin and 10 for the scalp, and mucosal involvement as 120. Thirteen points are also considered as damage score, which due to lack of representation of current disease status was not evaluated in the present study. Total PDAI activity score excluding damage score defined as the sum of cutaneous and mucosa scores.

The ABSIS is another relatively sensitive scoring system, proposed by the German Blistering Disease Group in 2007 [6], relies on the combination of both subjective and objective information. ABSIS system has a total score ranging from 0 to 206, consisting 150 points for skin involvement, 11 points for oral involvement, and 45 points for severity (discomfort during eating and drinking). In the analysis, the sum of scores for oral involvement and severity was considered as the mucosal score.

Physician’s global assessment (PGA) is a visual analogue scale, varies between the 0, as no lesion, and 10, representing disease severity [5]. PGA final scores were calculated as the mean of the PGA scores given to the patient by at least three dermatologists.

Autoimmune Bullous Disease Quality of Life (ABQoL) is a valid and reliable tool to determine the patient-reported outcome and quality of life in patients with autoimmune bullous diseases, containing 17-items questionnaires [10, 11]. In this study, ABQoL total score was calculated by adding up the first 16 questions of the questionnaire, since question #17 was left unanswered by 61 cases probably due their being unemployed, housekeeper, or owing independent businesses.

*ELISA testing of anti-desmoglein 1 and 3 antibodies* Serum sample was collected and anti-Dsg3 and anti-Dsg1 values were determined (EUROIMMUN Medizinische Labordiagnostika AG, Lübeck, Germany). Values greater than 20 U/ml were considered positive.

### Statistical analysis

Cut-off values were calculated by employment of model-based clustering and the 25th and 75th percentiles. In the former approach, model-based clustering classification was conducted using Gaussian finite mixture model via Mclust-package of R software to determine the number of clusters and subsequently define the patients’ category [12]. In the latter, which was used by Boulard et al. [13], regardless of distribution of data, three categories were defined as moderate (lower than 25th percentile), significant (equal to or higher than the 25th percentile and lower than 75th percentile), and extensive (equal or higher than 75th percentile). The median of PDAI scores were compared in disease groups categorized based on ABSIS score, and vice versa. Moreover, to validate the classification of the three defined subgroups, median PGA and ABQoL, were compared based on cut-off values calculated from the PDAI and the ABSIS scoring systems. Based on these classifications, we also aimed to set optimum cut-off value for anti-Dsg1 and anti-Dsg3 values via ROC curve analysis. According to the area under the curve (AUC) and youden’s index, the best cut-points were chosen. ANOVA analysis was done for investigating the significance of different scores’ difference among subgroups. To find the association between two continuous variables, Pearson correlation coefficient has been employed. All analysis was done using SPSS version 21 (IBM Corp. Released 2012. IBM SPSS Statistics for Windows, Version 21.0. Armonk, NY: IBM Corp.) and R software version 3.3.1. *P*-value less than 0.05 was considered statistically significant.

For validating the classification of disease severity according to PDAI and ABSIS, the difference of other severity scores including PDAI or ABSIS, PGA, and ABQoL was assessed among the subgroups.

## Results

### Patients characteristics

One hundred-nine PV patients (27 [24.8%] men and 82 [75.2] women) were included. The mean age was 45 ± 12.6 years (20–85). Fifteen patients (13.8%) were categorized as exclusive cutaneous involvement, 47 (43.1%) have only mucosal involvement, and 47 (43.1%) had mucocutaneous involvement.

The subjects’ PDAI activity and ABSIS scores ranged from 1 to 84, and 0.1 to 88.5 distributed over the first 34 and 43% of the scales with the median of 13 and 15.1, respectively. As presented in Table 1, the median of PGA and ABQoL scores were 3.33 and 31, respectively. PDAI score was positively and significantly correlated with ABSIS (r = 0.636, *P*-value < 0.001), PGA score (r = 0.823, *P*-value < 0.001), and anti-Dsg1 value (r = 0.457, *P*-value < 0.001), while were negatively and significantly correlated with ABQoL (r = − 0.385, *P*-value < 0.001). ABSIS was also correlated positively with PGA score (r = 0.592, *P*-value < 0.001) and negatively with ABQoL score (r = − 0.269, *P*-value = 0.01), both significantly. Notable, unlike PDAI, ABSIS was positively and significantly correlated with anti-Dsg3 values (r = 0.218, *P*-value = 0.024) but not anti-Dsg1 values (r = 0.073, P-value < 0.456). PGA score was correlated with all the other scores and values significantly, except anti-Dsg3; but negatively correlated with ABQoL (r = − 0.486, P-value < 0.001).

**Table 1:** Median scores in different pemphigus subgroups

Disease site	PDAI			ABSIS			PGA	ABQoL
	cutaneous score*	Mucosal score	Total activity score	cutaneous score	Mucosal score**	Total activity score		
All patients (IQR)	2 (14)	6 (10)	13 (21.4)	0.1 (3.1)	12.5 (26.5)	15.1 (26)	3.33 (2.83)	31 (15.5)
Cutaneous (IQR)**	13 (12.9)	0	13 (12.9)	3.17 (25.99)	0	3.175 (25.99)	3.16 (4.21)	24.5 (22)
Mucosal (IQR)	0	7 (9.2)	7 (9.2)	0	17.5 (28.88)	17.5 (28.88)	2 (1.58)	36 (5.5)
Mucocutaneous (IQR)	10 (23.8)	6.3 (9)	20 (24.5)	2 .25 (6.76)	15 (25.5)	20.55 (22.86)	4.16 (3)	26 (14)

*Sum of skin and scalp scores for PDAI score

**Sum of scores for oral involvement and severity was considered as mucosal score

*Abbreviation*: *PDAI* pemphigus disease area index, *ABSIS* Autoimmune Bullous Skin Disorder Intensity Score, *ABQoL* autoimmune bullous disease quality of life, *PGA* physician global assessment, *IQR* interquartile range

### Cut-off values for disease severity scores based on the 25th and 75th percentiles

Categorizing patients based on the first and third quartiles resulted in moderate (less than first quartile score), significant (equal or higher than first quartile score and less than third quartile score), and extensive (higher than third quartile score) groups. The cut-off values for total PDAI activity score, obtained from quartile method, were 6.2 and 27 for all patients, 8.25 and 21.15 for patients with only cutaneous involvement, 3.6 and 20 for exclusive mucosal involvement, and 9.3 and 33.4 for patients with both cutaneous and mucosal lesions. The ABSIS scoring system cut-points were 4 and 29.5 for all patients, 2.07 and 11.94 for exclusive cutaneous patients, 3 and 31.63 for patients with only mucosal involvement, and 12.3 and 33.65 in mucocutaneous patients.

### Clustering-model-based cut-off values for disease severity scores

**Fig. 1:**
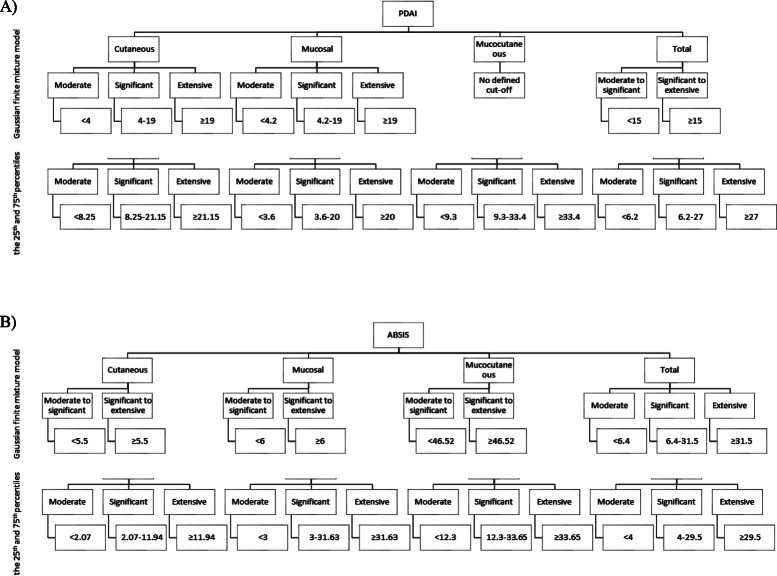
Suggested quartile and model-based categorizing groups for pemphigus severity in each subtype based on Pemphigus Disease Area Index (PDAI) **a** and Autoimmune Bullous Skin Disorder Intensity Score (ABSIS) **b**

According to model-based analysis for PDAI score in each subgroup, the calculated cut-points were 4 and 19 for the cutaneous group and 4.2 and 19 for the mucosal group and in contrast, 15 for total patients (Fig. 1a). According to the PDAI scoring system, no cut-point was suggested for mucocutaneous group. Regarding ABSIS score, each of the subgroups was divided into two categories. Calculated cut-points were 5.5 for the cutaneous group, 6 for the mucosal group, and 46.52 for the mucocutaneous group. However, total ABSIS score was divided into three categories of moderate (< 6.4), significant (6.4–31.5), and extensive (> 31.5) (Fig. 1b).

### Desmoglein cut-off values based on disease severity scores

**Fig. 2:**
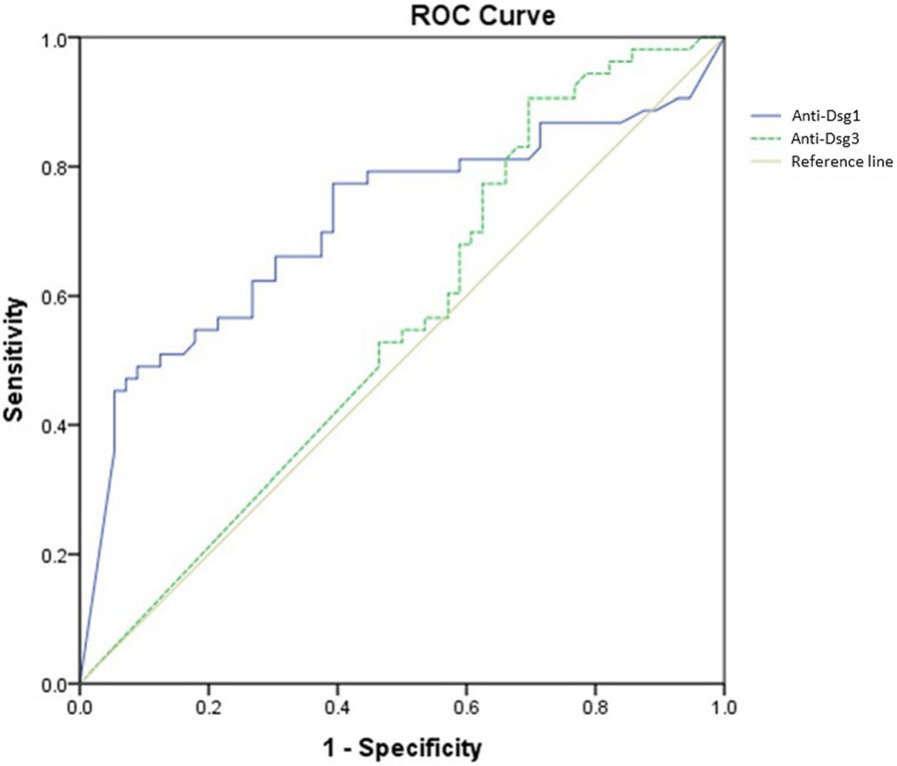
Receiver operating characteristic curve (ROC) curve analysis; showing the predictive power of anti-Dsg1 (desmoglein) and anti-Dsg3 values to define patients into two groups of moderate-to-significant and significant-to-extensive based on Pemphigus Disease Area Index (PDAI)-derived severity groups

When analyzing the anti-Dsg1 and anti-Dsg3 values in each categorized group, anti-Dsg1 values were highly correlated with disease severity by PDAI, but not ABSIS scores in patients with exclusive cutaneous involvement and total patients. Interestingly, these associations were observed after either performing model-based analysis or subgrouping according to quartiles. According to the model-based clustering in total patients suggested 128 U/ml as cut-point for categorizing patients into moderate-to-significant, and significant-to-extensive groups. Figure 2 shows the predictive power of anti-Dsg1 and anti-Dsg3 values to define patients into these two groups, moderate-to-significant, and significant-to-extensive groups based on total PDAI score in total patients, via ROC curve analysis. Additionally, quartile analysis resulted in cut-point values of 179 U/ml and 182.5 U/ml for total patients. Regarding anti-Dsg3 values, no association with disease severity based on PDAI and ABSIS scores was noted. Table 2 summarizes the details regarding categories of patients, according to the anti-Dsg1 values in patients with exclusive cutaneous involvement and total patients.

**Table 2:** Defined categories for prediction of defined disease severity by PDAI scores in total patients according to the anti-Dsg1 values (U/ml)

Patients	Groups			Anti-Dsg1 based categorization characteristics		
	Moderate	Significant	Extensive	Sensitivity	Specificity	AUC
Total patients (*n* = 109) *	Moderate-to-significant: < 128; Significant-to-extensive: > 128			0.49	0.91	0.71
Total patients (*n* = 109) **	< 179	179–182.5	> 182.5	0.33	1.00	0.68
				0.63	0.89	0.74

Cut-off values are calculated based on *model-based clustering analysis and **25th and 75th percentiles

*Abbreviations: PDAI* pemphigus disease area index, *anti-Dsg1* anti-desmoglein 1, *AUC* area under the curve

### Validation of the classification of severity subgroups

**Fig. 3:**
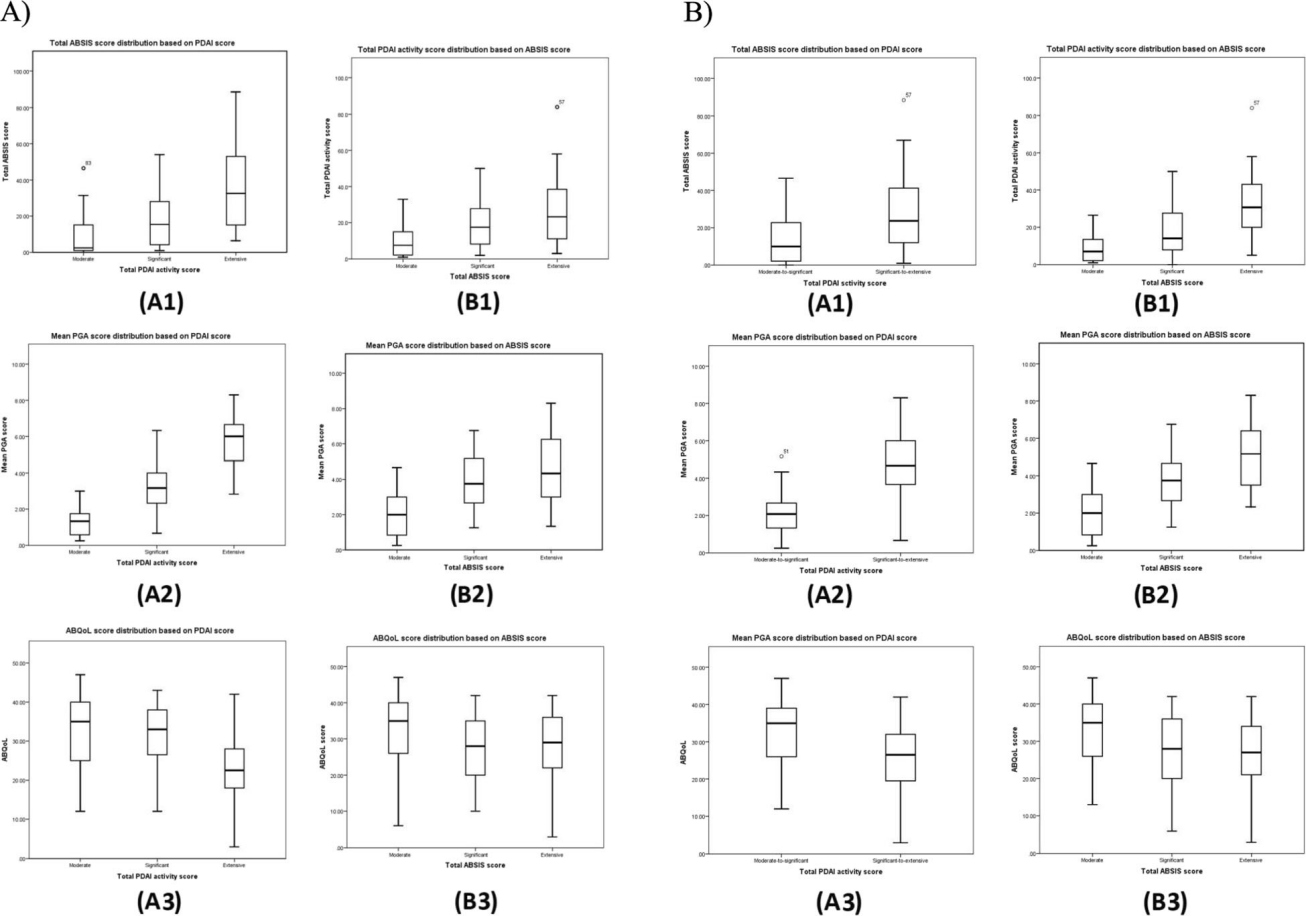
Boxplot of distribution of (A1) Autoimmune Bullous Skin Disorder Intensity Score (ABSIS), (A2) Physician’s Global Assessment (PGA) scores, and (A3) the Autoimmune Bullous Disease Quality of Life (ABQoL) score based on Pemphigus Disease Area Index (PDAI) activity score three extent subgroups; and (B1) Pemphigus Disease Area Index (PDAI) activity score, (B2) Physician’s Global Assessment (PGA) scores, (B3) the Autoimmune Bullous Disease Quality of Life (ABQoL) score based on Autoimmune Bullous Skin Disorder Intensity Score (ABSIS) three extent subgroups according to quartiles(I) and model-based analysis(II)

After classifying patients according to the PDAI and ABSIS scores cut-offs, based on percentile and model-based clustering methods, all other scores were significantly different among subgroups consisted of PDAI or ABSIS, PGA, and ABQoL, presented in Fig. 3.

## Discussion

In this study, we have suggested cut-off values for PDAI and ABSIS scoring systems based on either quartiles or models-based clustering. Regarding 25th and 75th percentiles, cut-off values were found 6.2 and 27 for the total PDAI activity score, and 4 and 29.5 for total ABSIS score. In model-based clustering, two groups with the cut-point of 15 were suggested for total PDAI activity score and three groups with cut-points of 6.4 and 31.5 for total ABSIS score.

Newer scoring systems are designed to present the disease activity and severity quantitatively. However, defining severity categories based on these scoring systems is needed in different clinical situations for specified medical and non-medical management. Severity categories were defined in previous scoring systems primarily based on expert opinion. Newer quantitative scoring systems (PDAI and ABSIS) enabled us to set cut-off points via statistical methods. Two studies have previously presented cut-off points for PDAI and/or ABSIS systems. Shimizu et al. [14] evaluated 37 pemphigus patients’ PDAI scores via analyzing maximum difference between sensitivity and 1-specifity, with their results being almost the same as ours. Boulard et al. [13] reported severity scores for a higher number of patients, suggesting three categories of moderate, significant, and extensive based on 25th and 75th percentiles. In the present study, with the percentile approach, PDAI score cut-offs were lower than Boulard et al.’s [13] (6.2 and 27 vs. 15 and 45) and closer to Shimizu et al.’s [14] (9 and 24) figures. Regarding ABSIS score, we reported lower cut-points than Boulard et al. [13] (4 and 29.5 vs 17 and 53), even with model-based clustering analysis with cut-offs of 6.4 and 31.5. Boulard et al. [13] included newly diagnosed treatment-naïve pemphigus patients, therefore, our lower cut-offs are probably caused by the less severe disease in our patients.

Using the quartiles for categorization will result in three subgroups which might not be necessarily consistent with clinical findings and actual severity of the disease; model-based analysis estimated two groups for total PDAI activity scores. However, the number of subgroups were the same in the percentile approach and model-based clustering in patients with exclusive cutaneous and exclusive mucosal involvement. Another notable difference in each approach was the detection of only one category in a model-based analysis comparing to three categories based on quartiles in patients with mucocutaneous involvement. Regarding total ABSIS scores, the two approaches suggested almost similar cut-points. In contrast to cut-off values for total ABSIS score, calculated values by model-based and quartiles analyses were different for each of cutaneous, mucosal, and mucocutaneous groups. Additionally, anti-Dsg1 presented valid cut-off values according to total PDAI scores for all the patients and exclusive cutaneous group. Overall, despite different results, both approaches have estimated validated cut-off values for categorizing patients, confirmed with the significant difference in the total PDAI activity or total ABSIS scores, PGA values, and ABQoL in each defined group.

Another important finding in our study was that unlike anti-Dsg1 values, anti-Dsg3 values were not able to be used as an indicator of the disease activity in the studied sample. To elaborate on, anti-Dsg1 values, but not anti-Dsg3, were associated with disease activity –based on PDAI and ABSIS– among our pemphigus patients. Anti-Dsg1 and anti-Dsg3 antibodies are both well-studied markers for pemphigus [15–18]. However, some evidence implies that anti-Dsg1 might be even more representative of the clinical course [8, 15, 19]. Indeed, some studies showed anti-Dsg1 antibody correlates with disease severity and even could be used as a predictor for tapering corticosteroid dosage after rituximab administration [15, 16, 20]. These findings probably could be explained by the presence of non-pathogenic anti-Dsg3 antibodies in the serum of patients [15, 18, 21]. As previously reported, there are some non-pathogenic antibodies that bind to the epitopes of Dsg3 [22]. In the conventional ELISA assay, both pathogenic and non-pathogenic anti-Dsg3 antibodies are detected, which might cause the overestimation of pathogenic anti-Dsg3 antibodies. The presence of non-pathogenic anti-Dsg3 could also help in understanding the observed high values of anti-Dsg 3 in patients with complete remission [15, 18, 21], whereas anti-Dsg1 antibodies correlated with disease activity and was a good predictor of relapse when present [15]. A large part of anti-Dsg3 antibodies in this situation could be considered non-pathogenic. Besides, the non-pathogenicity of these antibodies in patients in remission was further demonstrated in keratinocytes dissociation assays in vitro [23]. Finally, the observed pattern could be the result of a specific distribution of anti-Dsg3 measures in our sample, which could be furtherly improved with larger sample sizes.

To note, EDTA-based ELISA was proposed to distinguish between pathogenic and non-pathogenic anti-Dsg3, through measuring detection of non-calcium-dependent Dsg3 epitopes directed against the Dsg3. However, there are some controversies regarding the association between the estimated values of pathogenic anti-Dsg3 antibodies in this assay and disease severity [23–25] According to some findings, it seems that IgG4 is considered the most pathogenic subclass of IgG antibodies in PV [17, 26–28]. Thereby, the detection of only IgG4-specific antibodies against the Dsg3 might be another approach to make the anti-Dsg3 value a more useful marker for disease severity.

The current study has several limitations. Although we have suggested cut-points for classification of pemphigus severity through two different approaches, any patient with active lesion regardless of their treatment were recruited. This has led to the inclusion of the mildest cases seen in everyday practice. Moreover, relatively low number of patients might have caused some inaccuracy, as previously described.

## Conclusions

In conclusion, the classification of patients into subgroups of severity based on PDAI and ABSIS scores alongside with anti-Dsg1 value could help with managing patients more specified and efficient. Further studies are required including homogenous sample of patients from both disease subtype and treatment aspects. Additionally, higher number of patients for analysis for result in generalizable cut-off values. To note, current categories could help dermatologists for managing patients based on the severity of their disease and its specified medical and non-medical treatment plans, as well as monitoring the disease process.

## Data Availability

The data is available upon request by proposal to the corresponding author and permission of the funding institute. The funding institute had no role in the design of the study, collection, analysis, and interpretation of the data, and in writing the manuscript.

## Abbreviations

PV: Pemphigus vulgaris
PF: Pemphigus foliaceus
Anti-Dsg: Anti-desmoglein auto-antibody
PGA: Physician Global Assessment
PDAI: Pemphigus Disease Area Index
ABSIS: Autoimmune Bullous Skin Disorder Intensity Score
PVAS: Pemphigus Vulgaris Activity Score
PGA: Physician’s global assessment
ABQoL: Autoimmune Bullous Disease Quality of Life
